# Preparative Separation and Purification of Four Glycosides from *Gentianae radix* by High-Speed Counter-Current Chromatography and Comparison of Their Anti-NO Production Effects

**DOI:** 10.3390/molecules22112002

**Published:** 2017-11-17

**Authors:** Bao Chen, Yinghua Peng, Xinhui Wang, Zhiman Li, Yinshi Sun

**Affiliations:** 1 Institute of Special Animal and Plant Sciences, Chinese Academy of Agricultural Sciences, Changchun 130112, China; chenbao2333@163.com (B.C.); pengyinghua@caas.cn (Y.P.); wangxinhui.1989@163.com (X.W.); lzm091215@163.com (Z.L.); 2 College of Chinese Material Medicine, Jilin Agricultural University, Changchun 130118, China

**Keywords:** glycosides, *Gentianae radix*, high-speed counter-current chromatography (HSCCC), anti-NO production effects, nitric oxide production

## Abstract

Secoiridoid and iridoid glycosides are the main active components of *Gentianae radix*. In this work, one iridoid and three secoiridoid glycosides from *Gentianae radix* have been purified by high-speed counter-current chromatography in two runs using different solvent systems. Ethyl acetate–*n*-butanol–water (2:1:3, *v*/*v*/*v*) was the optimum solvent system to purify ca. 4.36 mg of loganic acid, 3.05 mg of swertiamarin, and 35.66 mg of gentiopicroside with 98.1%, 97.2% and 98.6% purities, respectively, while 31.15 mg of trifloroside with 98.9% purity was separated using hexane–ethyl acetate–methanol–water (1:3:1:3, *v*/*v*/*v*/*v*). The structures of the glycosides were identified by mass spectrometry and NMR. After separation, the anti-nitric oxide production effects of the compounds on lipopolysaccharide-induced BV-2 murine microglial cells were also evaluated. All of the compounds inhibited the production of nitric oxide in lipopolysaccharide-induced BV-2 cells with high cell viabilities in a concentration-dependent manner, which demonstrated that were able to be used as a nitric oxide inhibitor.

## 1. Introduction

*Gentianae radix*, the root and rhizome of *Gentiana scabra* Bunge, is officially listed as “Longdan” in the Chinese Pharmacopoeia [1]. It has been widely used to eliminate damp heat and quench the fire of the liver and gall bladder [2]. Secoiridoid and iridoid glycosides, such as gentiopicroside [3], swertiamarin [4], loganin [5], and loganic acid [6], are the major bioactive components of *G. radix* and secoiridoid glycosides directly related to its bitterness. Secoiridoid and iridoid glycosides are widely distributed in the plant kingdom, and have been extensively studied for their various pharmacological functions, such as hepatoprotective effects [7,8], muscle relaxing activity [9], analgesic activities [10], and anti-diabetic effects [11]. Due to these significant bioactivities, large quantities of pure compounds are urgently needed as reference standards and for various in vitro and in vivo studies related to the use of secoiridoid and iridoid glycosides.

High-speed counter-current chromatography (HSCCC) is an advanced technique based on a liquid–liquid partitioning. In this technique, there is no irreversible adsorption to the column. At the same time, it has the advantages of having a wide application range, simple operation, and large injection volume [12,13]. For these reasons, HSCCC has been used to separate secoiridoid and iridoid glycosides in several studies, such as the HSCCC separation using chloroform/methanol/water [14,15], *n*-butanol/ethyl acetate/methanol/water [16], and *n*-butanol/water solvent systems [17]. In this article, we have discussed the development of the HSCCC method for the separation and purification of loganic acid (1); swertiamarin (2); gentiopicroside (3); and trifloroside (4) from *G. radix*.

Several studies have shown that loganic acid, swertiamarin, gentiopicroside, and trifloroside have anti-inflammatory effects, as illustrated by some models of experimental inflammation, such as the lipopolysaccharide (LPS)-induced macrophage RAW 264.7 cells [18], dextran sulfate sodium-induced colitis in mice [19], etc. However, to the best of our knowledge, the effects of these four glycosides on the LPS-induced BV-2 murine microglial cells model have not been reported as yet. In the present study, the LPS-induced BV-2 murine microglial cells model was used to evaluate the nitric oxide (NO) production inhibitory activity of these four glycosides.

## 2. Results and Discussion

### 2.1. Optimization of HPLC Conditions

Several elution systems were tested for the HPLC separation of S_1_ and S_2_ (the extract of *Gentianae radix* was further extracted by distilled water and ethyl acetate (1/1, *v*/*v*), the concentrated water fraction was evaporated to dryness under reduced pressure to give sample 1 (S_1_) and ethyl acetate fraction was dried up to give sample 2 (S_2_)), such as gradient elution of methanol/water, acetonitrile/water, and methanol/acetonitrile/water. When the mobile phase was acetonitrile and water, good results were obtained for both S_1_ and S_2_. Other factors such as the detection wavelength (relatively higher absorption) and flow rate were also investigated. The best results were obtained with 254 nm and 1.0 mL/min as the detection wavelength and flow rate, respectively. Samples S_1_, S_2_, and the peak fractions separated by HSCCC were analyzed by HPLC under the optimized analytical conditions.

### 2.2. HSCCC Separation

A successful separation largely depends upon the selection of suitable two-phase solvent system, which can be quickly done by determining the partition coefficient (*K*) in the range of 0.5–2.0 [20,21,22]. Several two-phase solvent systems were tested and the *K* values were measured and summarized in Table 1.

**Figure 1:**
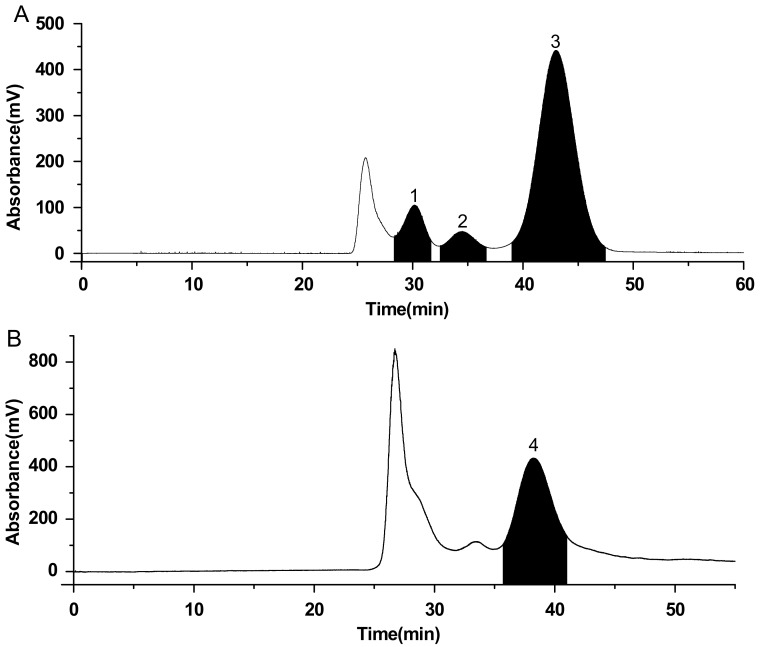
HSCCC separation chromatograms of samples obtained from *Gentianae radix*. (**A**) Sample 1, ethyl acetate–*n*-butanol–water (2:1:3, *v*/*v*/*v*); and (**B**) Sample 2, hexane–ethyl acetate–methanol–water (1:3:1:3, *v*/*v*/*v*/*v*); sample size = 100 mg; flow rate = 5 mL/min; detection wavelength = 254 nm; revolution speed = 800 rpm.

For the separation of compounds **1**–**3** present in S_1_, the *K* values of compounds **1** and **2** in the two-phase solvent system of ethyl acetate–*n*-butanol–water (4:1:5, *v*/*v*/*v*) was too small, as shown in Table 1, while the *K* values of compound **2** in the hexane-ethyl acetate-methanol-water (1:1:1:1, *v*/*v*/*v*/*v*), hexane–ethyl acetate–methanol–water (1:3:1:3, *v*/*v*/*v*/*v*) and hexane–ethyl acetate–methanol–water (1:4:1:4, *v*/*v*/*v*/*v*) were too large. Finally, the system of ethyl acetate–*n*-butanol–water (2:1:3, *v*/*v*/*v*) was selected because of its more suitable *K* values. Although *K*_1_ was somewhat small, Figure 1A shows that compounds **1**–**3** were well-separated with acceptable resolution.

For the separation of compound **4** in sample 2, it was found that the values of *K*_4_ corresponding to ethyl acetate–*n*-butanol–water (4:1:5, *v*/*v*/*v*), ethyl acetate–*n*-butanol–water (2:1:3, *v*/*v*/*v*), and hexane–ethyl acetate–methanol–water (1:4:1:4, *v*/*v*/*v*/*v*) solvent systems were too large, that of the K_4_ of hexane–ethyl acetate–methanol–water (1:1:1:1, *v*/*v*/*v*/*v*) too small, and the solvent system of hexane–ethyl acetate–methanol–water (1:3:1:3, *v*/*v*/*v*/*v*) gave an ideal *K* value and resulted in a good separation of compound **4** (Figure 1B). Therefore, hexane–ethyl acetate–methanol–water (1:3:1:3, *v*/*v*/*v*/*v*) was selected as the most suitable two-phase solvent system to separate compound **4**.

The optimization of other factors such as the resolution speed of the separation column, detection wavelength, and flow rate of the mobile phase was also performed. It was found that an appropriate retention percentage of the stationary phase and good separation results could be obtained at a resolution speed of 800 rpm, detection wavelength of 254 nm, and flow rate of 5 mL/min.

**Figure 2:**
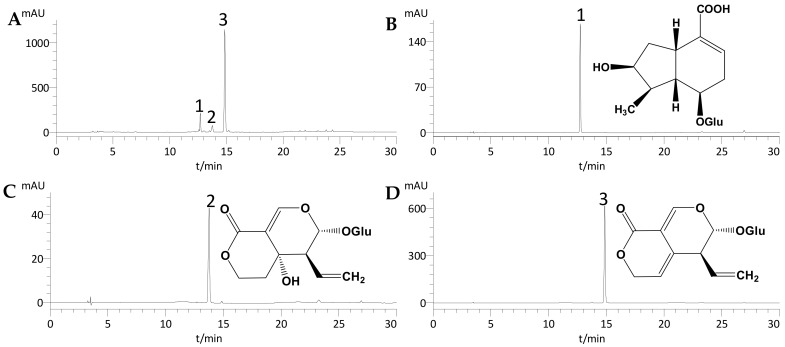
HPLC chromatograms of sample 1 and compounds **1**–**3** separated by HSCCC. (**A**) HPLC chromatogram of Sample 1; (**B**) HPLC chromatogram of compound **1**; (**C**) HPLC chromatogram of compound **2**; and (**D**) HPLC chromatogram of compound **3**.

**Figure 3:**
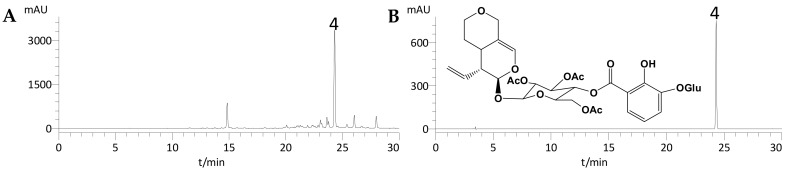
HPLC chromatograms of (**A**) sample 2 and (**B**) compound **4** separated by HSCCC.

Under the optimized conditions, Figure 1A showed that S_1_ was successfully separated with the two-phase solvent system of ethyl acetate–*n*-butanol–water (2:1:3, *v*/*v*/*v*) in 50 min. In total, 4.36 mg of loganic acid (compound **1**), 3.05 mg of swertiamarin (compound **2**), and 35.66 mg of gentiopicroside (compound **3**) were obtained from 100 mg of S_1_ with purities of 98.1%, 97.2% and 98.6%, respectively, as determined by HPLC (Figure 2). Figure 1B shows that S_2_ was also successfully separated with the two-phase solvent system of hexane-ethyl acetate-methanol-water (1:3:1:3, *v*/*v*/*v*/*v*) in about 45 min. From 100 mg of S_2_, 31.15 mg of trifloroside (compound **4**) was obtained with 98.9% purity (Figure 3).

In this study, four glycosides of high purity were separated by HSCCC from *Gentianae radix*. Adequate compounds for in-vitro pharmacological activity experiments were obtained in a one-step separation, demonstrated that HSCCC is a powerful and efficient tool for the separation of glycosides.

### 2.3. Identification of the Isolated Compounds

The purities of the four isolated compounds were over 96% (as determined by HPLC), and their structures were characterized by HPLC, MS, ^1^H-NMR, and ^13^C-NMR (data given below). A comparison of the HPLC retention times and MS, ^1^H-NMR, and ^13^C-NMR data with those described in previous reports led us to conclude that compounds **1**–**4** are loganic acid, swertiamarin, gentiopicroside, and trifloroside, respectively.

Compound **1**: ESI–MS *m*/*z*: 375.3 [M − H]^−^; ^1^H-NMR (400 MHz, DMSO-*d*_6_) *δ*: 5.09 (1H, d, *J* = 4.8 Hz, H-1), 7.30 (1H, s, H-3), 2.95 (1H, m, H-5), 1.42 (1H, m, H-6a), 1.79 (1H, m, H-6b), 3.87 (1H, m, H-7), 1.69 (1H, m, H-8), 2.05 (1H, m, H-9), 0.98 (1H, d, *J* = 6.7 Hz, H-10), 4.47 (1H, d, *J* = 7.8 Hz, H-1′). ^13^C-NMR (400 MHz, DMSO-*d*_6_): see Table 2. On comparison with the data reported by Calis et al. [23], compound **1** was identified as loganic acid.

Compound **2**: ESI–MS *m*/*z*: 397.2 [M + Na]^+^; ^1^H-NMR (400 MHz, DMSO-*d*_6_) *δ*: 5.59 (1H, d, *J* = 1.4 Hz, H-1), 7.52 (1H, s, H-3), 1.73 (2H, m, H-6a, H-6b), 4.26 (1H, m, H-7a), 4.56 (1H, m, H-7b), 5.29 (1H, d, *J* = 4.8 Hz), 2.82 (1H, m, H-9), 5.23 (1H, m, H-10a), 5.36 (1H, m, H-10b), 4.45 (1H, d, *J* = 7.8 Hz, H-1′), 2.82 (1H, t, H-2′), 3.15 (1H, m, H-3′), 3.03 (1H, m, H-4′), 3.15 (1H, m, H-5′), 3.66 (1H, m, H-6′a), 3.41 (1H, m, H-6′b). ^13^C-NMR (400 MHz, DMSO-*d*_6_): see Table 2. Compound **2** was identified as swertiamarin on comparison with the data given by Rana et al. [24].

Compound **3**: ESI–MS *m*/*z*: 379.2 [M + Na]^+^; ^1^H-NMR (400 MHz, DMSO-*d*_6_) *δ*: 5.59 (1H, d, *J* = 4.0 Hz, H-1), 7.41 (1H, s, H-3), 5.64 (1H, m, H-6), 5.07 (1H, m, H-7a) 4.94 (1H, m, H-7b), 5.67 (1H, m, H-8), 3.15 (1H, m, H-9), 5.18, 5.22 (2H, m, H-10), 4.49 (1H, d, *J* = 8.0 Hz, H-1′). ^13^C-NMR (400 MHz, DMSO-*d*_6_): see Table 2. On the basis of the data previously reported by Hajimehdipoor and co-workers [25], compound **3** was identified as gentiopicroside.

Compound **4**: ESI–MS *m*/*z*: 805.4 [M + Na]^+^; ^1^H-NMR (400 MHz, DMSO-*d*_6_) *δ*: 7.52 (1H, d, *J* = 2.1 Hz, H-3), 7.34 (1H, dd, *J* = 19.6, 8.4 Hz, H-4′′), 7.22 (1H, d, *J* = 8.0 Hz, H-6′′), 6.82 (1H, t, *J* = 8.0 Hz, H-5′′) 2.00 (3H, s, AcO), 1.90 (3H, s, AcO), 1.86 (3H, s, AcO). ^13^C-NMR (400 MHz, DMSO-*d*_6_): see Table 2. A comparison of this data with that reported by Kim et al. [26], led to the conclusion that compound **4** was trifloroside.

### 2.4. Inhibition of LPS-Induced NO by Glycosides

**Figure 4:**
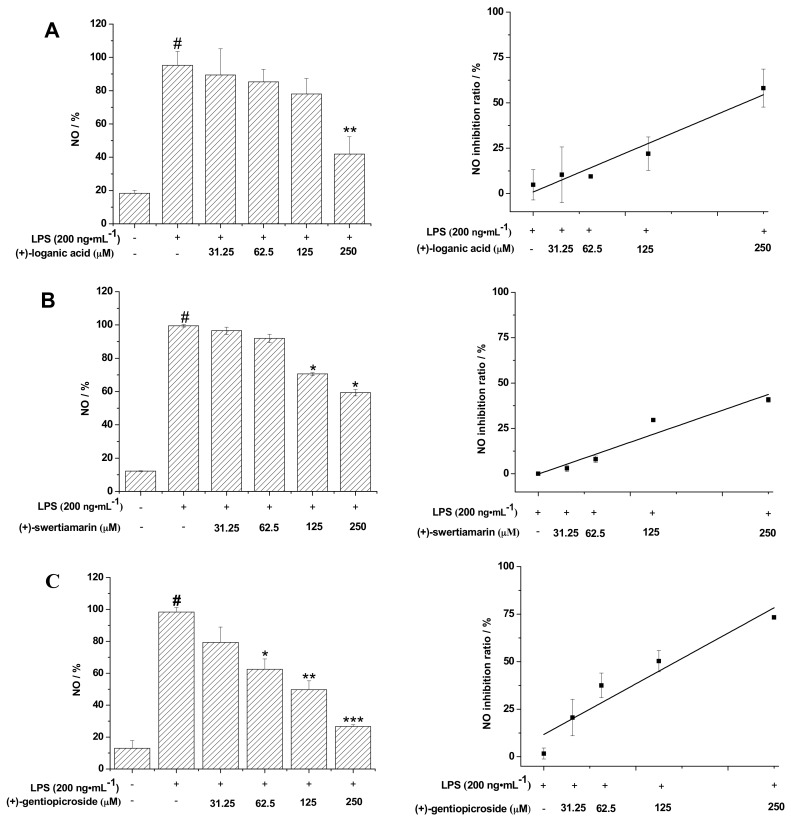

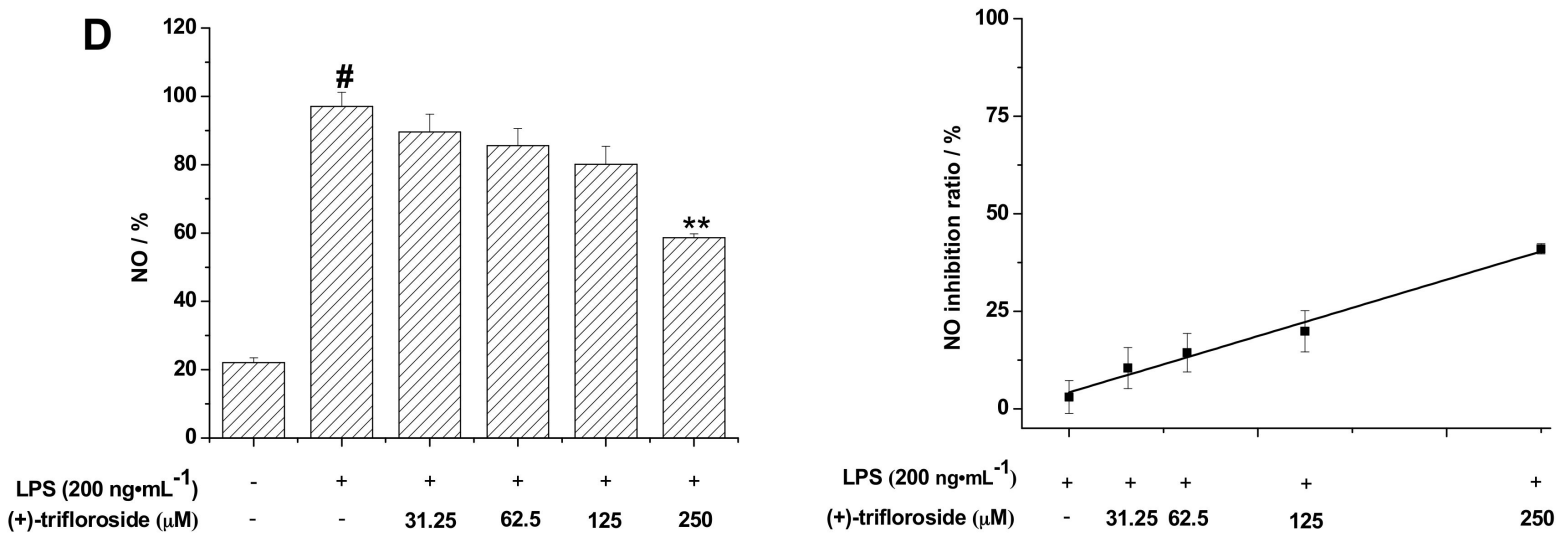
Inhibition of LPS-induced NO in microglial BV-2 cells and the NO inhibition rate of (**A**) loganic acid; (**B**) swertiamarin; (**C**) gentiopicroside; and (**D**) trifloside. BV-2 cells were treated with the indicated concentrations of (±)-glycosides and LPS (200 ng/mL) for 24 h. The NO content in the media was measured and the amount of NO in the LPS (200 ng/mL) group was set as 100%. The data is presented as the mean ± SD from three independent experiments and the differences between the mean values were assessed by the Student’s *t*-test: # *p* < 0.05 compared to the control group; * *p* < 0.05, ** *p* < 0.01, and *** *p* < 0.001 compared to the LPS group.

LPS, a main component of the outer membrane of gram-negative bacteria, can induce inflammation in cells. NO is produced from the amino acid l-arginine by the members of the NO synthase family of proteins, and is involved in several cellular functions, including neurotransmission, regulation of blood-vessel tone, and the immune response [27], and its level indicates the degree of inflammation [28]. After the cells were treated for 24 h, 100 μL of the supernatant media was collected to detect the concentration of NO. It was found that the level of NO gradually decreased in the concentration-dependent manner of gentiopicroside. The compound at the concentration of 250 µM significantly inhibited NO generation (73.3 ± 1.1%) (Figure 4C). Nitric oxide production was also suppressed by loganic acid (58.1 ± 10.4%), swertiamarin (40.8 ± 1.3%) and trifolioside (41.0 ± 1.3%). All the results showed that loganic acid, swertiamarin, gentiopicroside, and trifloroside were able to effectively inhibit NO production induced by LPS (200 ng/mL) in a dose-dependent manner in BV-2 cells. This result is consistent with the previous reports that secoiridoid glycosides exerted moderate inhibiting activities on LPS-induced NO production in Raw 264.7 macrophages [29].

### 2.5. Effects of Glycosides on Cell Viability

**Figure 5:**
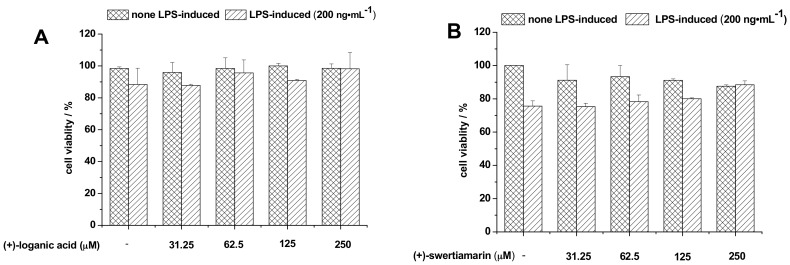

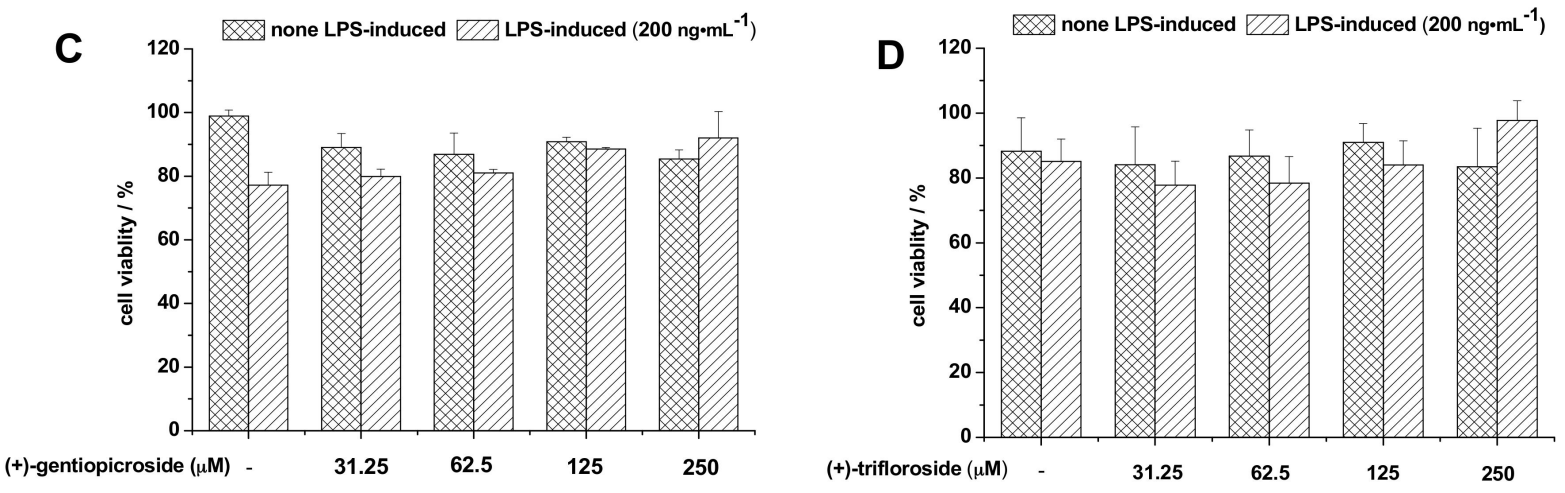
Effect of (**A**) longanic acid; (**B**) swertiamarin; (**C**) gentiopicroside; and (**D**) trifloroside on the cell viability of microglial BV-2 cells in LPS-treated or non-LPS-treated. BV-2 cells were treated with various concentrations of (±)-glycosides for 24 h and cell viabilities were monitored by crystal violet staining. Data are presented as means ± SD (*n* = 3 in each group).

The potential cytotoxicities of loganic acid, swertiamarin, gentiopicroside, and trifloroside were evaluated by crystal violet staining after incubating the cells for 24 h in both the absence and presence of LPS. As the indicated concentrations of the four glycosides were added into the plate, the groups of the LPS-induced or non-induced cells showed no obvious change in comparison with the control group (Figure 5). The result showed that all these four glycosides exhibited no toxic effects in BV-2 cells, which is consistent with previous reports [30,31]. This result also indicated that the cell viabilities were not affected by the highest concentrations of the glycosides and cytotoxicity is not a factor in inhibiting NO production. Therefore, we are interested in determining the mechanism of inhibition of NO production by loganic acid, swertiamarin, gentiopicroside, and trifloroside and understanding the Toll-Like Receptor signaling pathway as the major pathway in neurogenic inflammation. In the future, we aim to examine whether the decline in the level of NO is associated with this pathway.

## 3. Experimental

### 3.1. Apparatus

The HSCCC instrument employed in this study is a model TBE-300C high-speed counter-current chromatograph (Tauto Biotech, Shanghai, China) with a total capacity of 315 mL. The HSCCC system was equipped with a model TBP-5002 constant flow pump (Tauto Biotech, Shanghai, China), 200D UV detector with a preparative flow cell (Tauto Biotech, Shanghai, China), model DC-0506 constant-temperature controller (Tauto Biotech, Shanghai, China), and an Easy chrom-1000 workstation (Beijing Boyikang Laboratory Instruments, Beijing, China).

The high-performance liquid chromatography equipment used was an Essentia (Shimadzu, Kyoto, Japan) system including an LC-16 pump and a SPD-M20A detector. The evaluation and quantification of the data were conducted on a LabSolutions Essentia workstation.

The ESI-MS (MS/MS) data were obtained by a Waters ACQUITY UPLC system and Waters XEVO TQ-S tandem quadrupole mass spectrometer (Waters, Milford, Singapore), while the ^1^H-NMR and ^13^C-NMR experiments were performed on an INOVA-400 NMR spectrometer (Varian Corporation, Palo Alto, CA, USA) using DMSO-*d*_6_ as a solvent.

### 3.2. Chemicals and Reagents

All organic solvents used for HSCCC were of analytical grade and purchased from Beijing Chemical Works (Beijing, China). Acetonitrile used for HPLC was of chromatographic grade and purchased from Thermo Fisher Scientific (Waltham, MA, USA). Water used was purified using a Wotepu water purifier (Sichuan Wortel Water Treatment Equipment Co., Ltd., Sichuan, China) before use. Dulbecco’s modified Eagle’s medium (DMEM), fetal bovine serum (FBS), penicillin, and streptomycin for cell cultures were obtained from Invitrogen-Gibco (Grand Island, NY, USA). LPS and 2,3-diaminonaphthalene were purchased from Invivogen (San Diego, CA, USA) and Sigma (St. Louis, MO, USA), respectively. *Gentiana scabra* Bunge was purchased from Beijing TongRenTang Pharmacy Store (China) and identified by Dr. Y. N. Zheng, Jilin Agricultural University (Jilin, China).

### 3.3. Preparation of the Crude Extract

First, 0.5 kg of powdered *G. radix* was extracted three times (for 0.5 h each) with 5 L of methanol by sonication. The extracts were combined, filtered, evaporated to dryness under reduced pressure, and then extracted by distilled water and ethyl acetate (1/1, *v*/*v*). The concentrated water fraction was evaporated to dryness under reduced pressure to give sample 1 (S_1_, 75.28 g) and ethyl acetate fraction was dried up to give sample 2 (S_2_, 14.08 g). Then, S_1_ and S_2_ were stored in a refrigerator (4 °C) and subjected to subsequent HPLC analysis and HSCCC separation.

### 3.4. Selection and Preparation of a Two-Phase Solvent System for HSCCC

The two-phase solvent system was selected according to the partition coefficients (*K*) of the target components in S_1_ and S_2_. The *K* values were determined by HPLC analyses as follows: Approximately 10 mg of the samples were weighed into a 10 mL test tube and 4 mL of each phase of the equilibrated two-phase solvent system was added. The test tube was capped and shaken vigorously for several minutes to thoroughly equilibrate the sample between the two phases. Next, an equal volume (2 mL) of the upper and lower phases was separately evaporated to dryness. The residues were dissolved with methanol to 1 mL and analyzed by HPLC to determine the *K* values of loganic acid (*K*_1_), swertiamarin (*K*_2_), and gentiopicroside (*K*_3_) in S_1_, and trifloroside (*K*_4_) in S_2_. The peak area of the upper phase was recorded as *A_U_* (area of the upper phase) and that of the lower phase was recorded as *A_L_* (area of the lower phase). The *K* value was calculated according to the equation *K = A_U_*/*A_L_* [32,33].

### 3.5. HSCCC Separation Procedure

The two-phase solvent system was prepared in a separation funnel and thoroughly equilibrated for 24 h. Next, the upper and lower phases were separated and degassed by sonication for 30 min before use. The separation multilayer coiled column was first entirely filled with the upper organic phase (stationary phase). Next, the apparatus was rotated at a speed of 800 rpm, while the lower phase (mobile phase) was pumped into the inlet of the column at a flow rate of 5.0 mL/min. After a hydrodynamic equilibrium was achieved, as indicated by a clear mobile phase eluting at the tail outlet, S_1_ and S_2_ were separately dissolved in 10 mL of the mixture of the upper and lower phases (1:1, *v*/*v*) and injected into the separation column through the injection valve. The effluent from the column outlet was continuously monitored with a UV detector at 254 nm. Each peak fraction was manually collected according to the chromatogram, evaporated under reduced pressure, and analyzed by HPLC.

### 3.6. HPLC Analysis of the HSCCC Peaks

The samples (S_1_ and S_2_) and each fraction peak from HSCCC was analyzed by HPLC with a 5-μm ZORBAX SB-C18 column (4.6 mm × 150 mm, Agilent Technologies, Santa Clara, CA, USA). HPLC analysis was performed by using a mobile phase of water (A) and acetonitrile (B) mixture at a flow rate of 1.0 mL/min. Chromatograms were recorded at 254 nm and the gradient program for S_1_ and S_2_ was as follows: 5% B (0–5 min), 5–13% B (5–6 min), 13–20% B (6–14 min), 20–33% B (14–15 min), 33–55% B (15–25 min), and 55–100% B (25–30 min).

### 3.7. Activity of Reducing LPS-Induced NO Production in BV-2 Cells

#### 3.7.1. BV-2 Cell Culture

The murine microglial BV-2 cell line was provided by Dr. Rona Giffard (Stanford University, Palo Alto, CA, USA) and grown in supplemented DMEM (including 10% FBS, 50 unit/mL of penicillin, and 50 μg/mL of streptomycin). BV-2 cells were detached from the culture dish with a cell shovel when ca. 80% confluence was reached. The cells were seeded at a density of 3 × 10^5^ cells per well in 96-well plates. After overnight incubation, the media was aspirated and changed to DMEM media without FBS. The cells were then treated with the four glycosides at different concentrations, followed by stimulation with LPS (200 ng/mL). LPS induced ca. 90% of the maximal response at the specified concentrations.

#### 3.7.2. Nitric Oxide (NO) Assay

In this assay, 100 μL of supernatant media was first collected into flat black 96-well microfluor plates (Corning) after the cells had been treated with the indicated concentrations of (±)-glycosides (0, 31.25, 62.5, 125 and 250 μM) and LPS (200 ng/mL) for 24 h. Subsequently, 10 μL of the 2,3-diaminonaphthalene (0.05 mg/mL in 0.62 M HCl) was added to each well and incubated at room temperature for 15 min. Finally, the reaction was terminated by the addition of 5 μL of 3 M NaOH, and the plate was read on a SYNERGY H1 reader (BioTek Instruments Inc., Winooski, VT, USA) with the excitation at 360 nm and the emission at 430 nm refer to the report by Wang [34].

#### 3.7.3. Cell Viability Assay

Crystal violet staining was used to determine cell viability. Briefly, after treatment, the cells were fixed with 4% paraformaldehyde for 5 min and then stained with 0.05% crystal violet at room temperature for 15 min. Subsequently, the plates were washed three times with tap water and dried for 1 h at room temperature. This was followed by the addition of 200 μL of methanol to each well. Finally, the plates were shaken to dissolve the dye at room temperature for 15 min. Absorbance at 510 nm was measured on a BioTek SYNERGY H1 reader.

## 4. Conclusions

In this work, an effective HSCCC method was developed for the preparative isolation and purification of loganic acid, swertiamarin, gentiopicroside, and trifloroside from *Gentianae radix*. These results clearly demonstrated that HSCCC is a powerful and efficient tool for the separation of compounds derived from natural resources. Loganic acid, swertiamarin, gentiopicroside, and trifloroside were able to inhibit the production of NO in LPS-induced BV-2 cells effectively with high cell viabilities, which demonstrated that they were able to be usedas a NO inhibitor.

## Figures and Tables

**Table 1:** *K* values of the four compounds in different two-phase solvent systems.

Solvent Systems (*v*/*v*)	*K* Values			
	1	2	3	4
ethyl acetate–*n*-butanol–water (4:1:5)	0.22	0.35	0.54	11.92
ethyl acetate–*n*-butanol–water (2:1:3)	0.41	0.56	0.73	9.82
hexane–ethyl acetate–methanol–water (1:1:1:1)	0.49	9.00	0.00	0.01
hexane–ethyl acetate–methanol–water (1:3:1:3)	0.50	3.54	0.01	1.89
hexane–ethyl acetate–methanol–water (1:4:1:4)	0.55	2.95	0.17	3.78

Compound **1**, loganic acid; Compound **2**, swertiamarin; Compound **3**, gentiopicroside; and Compound **4**, trifloroside.

**Table 2:** ^13^C-NMR data of compounds **1**–**4** in DMSO-*d*_6_.

Position	Compounds			
	1	2	3	4
1	96.0	96.4	96.4	95.8
3	150.0	152.0	148.8	149.1
4	112.6	108.1	103.3	105.6
5	30.9	64.1	125.0	27.4
6	41.8	32.1	116.1	24.3
7	73.1	69.95	69.2	68.3
8	40.5	132.9	134.0	132.0
9	44.7	49.9	44.4	41.2
10	13.6	120.4	117.9	120.9
11	168.1	164.4	162.8	164.5
1′	98.5	98.3	98.8	96.8
2′	72.1	72.9	72.8	71.3
3′	76.8	77.4	77.4	70.8
4′	70.1	70.0	70.0	70.1
5′	77.2	76.1	76.6	71.7
6′	61.1	60.9	61.2	61.9
1′′				115.6
2′′				151.2
3′′				146.3
4′′				123.3
5′′				119.0
6′′				121.1
7′′				166.1
1′′′				102.0
2′′′				73.5
3′′′				76.3
4′′′				69.0
5′′′				77.5
6′′′				61.0
OCOCH_3_				170.3
				169.8
				169.1
OCOCH_3_				20.8
				20.6
				20.5
